# Toward Predicting Social Support Needs in Online Health Social Networks

**DOI:** 10.2196/jmir.7660

**Published:** 2017-08-02

**Authors:** Min-Je Choi, Sung-Hee Kim, Sukwon Lee, Bum Chul Kwon, Ji Soo Yi, Jaegul Choo, Jina Huh

**Affiliations:** ^1^ Department of Computer Science and Engineering Korea University Seoul Republic Of Korea; ^2^ Department of Industrial ICT Engineering Dong-eui University Busan Republic Of Korea; ^3^ Department of Industrial Engineering Purdue University West Lafayette, IN United States; ^4^ IBM Thomas J. Watson Research Center Yorktown Heights, NY United States; ^5^ Samsung Electronics Co., Ltd. Seoul Republic Of Korea; ^6^ Division of Biomedical Informatics University of California San Diego San Diego, CA United States

**Keywords:** online health social network, machine learning, gradient boosting trees, prediction models, social media, online health community

## Abstract

**Background:**

While online health social networks (OHSNs) serve as an effective platform for patients to fulfill their various social support needs, predicting the needs of users and providing tailored information remains a challenge.

**Objective:**

The objective of this study was to discriminate important features for identifying users’ social support needs based on knowledge gathered from survey data. This study also provides guidelines for a technical framework, which can be used to predict users’ social support needs based on raw data collected from OHSNs.

**Methods:**

We initially conducted a Web-based survey with 184 OHSN users. From this survey data, we extracted 34 features based on 5 categories: (1) demographics, (2) reading behavior, (3) posting behavior, (4) perceived roles in OHSNs, and (5) values sought in OHSNs. Features from the first 4 categories were used as variables for binary classification. For the prediction outcomes, we used features from the last category: the needs for emotional support, experience-based information, unconventional information, and medical facts. We compared 5 binary classifier algorithms: gradient boosting tree, random forest, decision tree, support vector machines, and logistic regression. We then calculated the scores of the area under the receiver operating characteristic (ROC) curve (AUC) to understand the comparative effectiveness of the used features.

**Results:**

The best performance was AUC scores of 0.89 for predicting users seeking emotional support, 0.86 for experience-based information, 0.80 for unconventional information, and 0.83 for medical facts. With the gradient boosting tree as our best performing model, we analyzed the strength of individual features in predicting one’s social support need. Among other discoveries, we found that users seeking emotional support tend to post more in OHSNs compared with others.

**Conclusions:**

We developed an initial framework for automatically predicting social support needs in OHSNs using survey data. Future work should involve nonsurvey data to evaluate the feasibility of the framework. Our study contributes to providing personalized social support in OHSNs.

## Introduction

The social support model [1,2] received substantial interest in the field of medical informatics. According to the model, social support consists of emotional [3] and informational [2] support; the latter can further be specified into experience-based information [4], unconventional information, and medical facts [5]. Online health social networks (OHSN) users exchange emotional support by encouraging and sympathizing with others. Experience-based information includes a user’s experience and feelings about previously tried treatments or diet, or symptoms one had to undergo while suffering from a specific illness [6-8]. Unconventional information, though similar to experience-based information, lacks scientific background and comprises more radical approaches and treatments [9]. Fresh information on upcoming medicine or treatments is also included in this category. Lastly, medical facts refer to traditional medical information, such as experiments and other statistical data as well as published writings on illnesses and treatments, such as a doctor’s online blog. Patients have their own social support needs, and correctly understanding social support needs and providing adequate measures has positive effects on patients of both mental and physical conditions [10-12].

This model further developed as the Internet became prevalent to the general public, with OHSNs (eg, PatientsLikeMe [13], WebMD [14], Diabetes Daily [15], and Facebook [9,16]) emerging as a scalable platform for social support and improving health behavior. One underlying reason of its success is the variety of generated contents available. Some OHSN users prefer not to interact with others and just get information, while others prefer to bond with other patients via the Internet [17,18]. Unlike blogs or websites, which are updated by a few moderators with sufficient expertise, content in OHSNs is generated by diverse users with different interests and knowledge levels. Posts and threads are created around interesting topics, which provide better feedback compared with the one-way information delivery inherent in conventional websites. Also, the type of support both requested and generated in OHSNs are not restricted to refined medical facts, but comprises a wide range varying from unscientific, radical experiments to reassurance from peers. Simply put, OHSNs provide excellent grounds for users to fulfill their social support needs.

However, because of this abundance of information, OHSNs also suffer from several problems regarding the sustainability of a community. Most OHSN users visit these social networks in hope of effectively finding information relevant to their immediate needs. Having to instead search through a plethora of text or not being able to receive any replies to questions lead to exhaustion and frustration, making users visit only sporadically and seldom return. This may lead to shortened retention, a common problem with any technology adoption [19]. Monotonous support services to users will increase attrition [20,21] due to the lack of personalization in the content and the support users are receiving, possibly causing user migration [22-24].

A possible solution for OHSNs to both serve as a social support platform as well as maintain users is to provide customized information on an individual basis. This seemingly idealistic task can be materialized by using the vast amount of data provided by OHSNs, such as user-generated postings, user logs (eg, page visit records), and profiles that we can use as potential predictive factors for understanding each user. User characteristics in OHSNs include users’ self-reported profiles, visiting frequency, contents of users’ posts, the posts users have read, and so on. With an increasing effort from hospital institutions providing OHSNs (eg, Mayo clinic [25], patient-powered research networks [16,26]), medical records data can further be used to predict users’ personalized social support needs. By applying state of the art machine learning and analysis techniques on these data sources, we can create a data-driven framework that accurately predicts users’ social support types and needs, and then provides useful information or advice based on such prediction results.

However, an unrefined prediction model based on all available data is likely to suffer from high computational cost as well as low accuracy. There is a strong need to identify which features are important for prediction. Also, such raw data is difficult to merge with previously obtained knowledge. In this aspect, surveys are more effective in understanding unknown user behavior as they can be designed based on previous understandings as well as pinpointing possible behavior types.

Thus, in this study, we developed a framework for predicting users’ social support needs using a more refined form of data from a carefully designed survey [17]. The survey was based on in-person interviews aimed to identify the most important aspects of OHSN user behaviors. We developed a prediction model using the survey outcomes and evaluated the results to discover which different data types potentially available in actual OHSNs best represent the behavioral aspects of known social needs. Our findings help channel our efforts toward data types critical in generating tailored support for OHSN users.

## Methods

### Data Collection and Processing

From our prior work, we conducted a Web-based survey about their activities and behaviors in OHSN use [17]. The survey consisted of 21 multiple-choice questions on a 5-point scale ranging from strongly disagree to strongly agree to corresponding statements and 4 open-ended questions. We used the survey results from our previous work [18]. We conducted interviews with OHSN users to identify various characteristics of user needs observed in OHSN and adapted the surveys based on existing validated social support inventories [27,28] and our interview results.

Survey targets were OHSN users interested in chronic diseases, including: HIV, cancer, diabetes, weight management, heart disease, attention deficit hyperactivity disorder, Parkinson’s disease, fibromyalgia, depression, and bipolar disorders. Previous results show that patients with chronic diseases increasingly seek more social support in OHSNs [29]. Because our intention was to predict social support needs, and because this is a first study to predict information needs based on meta information of OHSN use, we decided to scope our work within a previously known group of OHSN users who have increased social support needs—those interested in chronic illness.

We recruited participants from Web-based advertisements including Google and Facebook, and high traffic OHSNs (eg, reddit) suggested from the Google Ranking Algorithm, and we asked the participants to fill out the survey. A total of 184 participants, who have visited any OHSNs at least once in the past and were over 18- years old, responded to the survey.

We encoded the survey responses into a 184 × 38 matrix with 184 respondents, 34 features, and 4 outcome variables (see Multimedia Appendix 1). There were 21 multiple-choice questions with values assigned from 1 to 5, where 1 corresponds to the strongest level of disagreement and 5 to the strongest level of agreement toward the question. Two coders first discussed the coding scheme of grouping similar answers and assigning a categorical value for each group of answers using the first 10 responses of the 4 open-ended questions. Afterward, the coders independently coded all of the rest of the responses, and then compared their responses to reach an agreed result if there were any discrepancies. The resulting agreed results became our coding results

The survey questions included 5 categories: reading, posting, demographic, role, and values sought. The first 4 categories are indicators for collecting features of various user behaviors in OHSNs. These questions contain posting or reading preferences, one’s demographic status, self-perceived roles within a community, and so on. On the other hand, values sought questions contain information on the prediction outcome variables. To collect 4 prediction outcome variables on the 4 social support needs, we asked the following question: “The reason for visiting the online health support group is,” which had 4 multiple-choice questions asking whether users visit OHSNs to obtain emotional support (ExchangeEmo), experience-based information of others, (HearExp), unconventional information (GetUnusualInfo), or medical facts (SpecificSearch) (See Multimedia Appendix 1 for exact survey questions and corresponding responses).

To develop the 4 binary classifiers predicting social support needs, for each classifier, we assigned TRUE class values to the users who responded 5 (strongly agree) or 4 (agree) to each values sought question. The rest of the users were assigned a NEGATIVE class. For instance, if 1 respondent rated 5 for ExchangeEmo but 1 for HearExp, the user was classified as TRUE for the ExchangeEmo classifier and NEGATIVE for the HearExp classifier. We can assume that this person has a strong need for sharing emotional peer support, but is less inclined toward hearing from the experiences of others. The rationale behind this selection is that lowering the threshold of positive values to 3 dilutes the strength of characteristics inherent in features representing a specific social support need, while further increasing the threshold to separate scores of 4 and 5 increases bias. Also, excluding 3 from classification removes at least 53 of 184 training data samples (28.8%) of the training data (See Multimedia Appendix 1 – SpecificSearch). Therefore, this selection method was adopted to both preserve strong characteristics and data size.

### Classification Algorithms

We performed our classification task using a wide variety of machine learning algorithms, which have been heavily applied to binary classification. We selected gradient boosting tree (GBT) [30-32], support vector machines (SVM) [33], decision tree [34], random forest [35], and logistic regression [36] as classifiers to compare the evaluation results. We built 4 models (predicting each of the 4 social support needs) for each classifier.

We used the area under the receiver operating characteristic (ROC) curve (AUC) value as the performance measure [37]. A large AUC value over 0.8 denotes a reasonably good prediction rate [38], while an AUC value of 0.5 is equal to the predictability of a purely random output such as a coin flip. One advantage of adopting the AUC measure is that it is invariant of data imbalance. It measures how well positive data samples are ranked higher than negative samples, and produces reliable results even in positively or negatively skewed datasets. In addition, the AUC measure contains information on all possible precision and recall value pairs as it uses various threshold values of the classifier output about whether a value is positive or negative [39]. For these reasons, we used AUC throughout our experiments.

We included the correlations between the features and each outcome variable to report the direction of association missing in the AUC values (see Multimedia Appendix 3).

Each prediction model followed the following steps, where averaging the outcomes from multiple epochs was performed to compensate for the relatively small sample size:

1. Randomly split data into 70/30, where 70.1% (129/184) of samples are used as a training set and the remaining 29.9% (55/184) as a test set.
  Train on the training set from (1).
  Run predictions on the test set from (1), and compare the predicted scores with the true scores of the outcome variable.
  Calculate AUC score of the predictability shown in (3).
2. Iterate steps (1)~(4) 50 times, with each iteration producing an AUC score.
3. Average the resulting 50 AUC scores.

We then conducted multiple experiments on each outcome variable, using only 1 feature at a time. The AUC values here corresponded to the strength of an individual feature’s predictability toward each social support need.

We used 1 feature at a time to measure the prediction power of 1 single variable on the outcome, instead of finding the best subset of features for maximal prediction accuracy. While feature selection methods may improve overall prediction results, this method does not match our aim toward discovering which individual features contain high predictive powers. Furthermore, advanced machine learning algorithms, such as GBT, random forests, and others, can properly use highly important variables while ignoring unimportant variables, given a large number of variables.

All the data training and prediction processes were performed with Matlab R2015a on an Intel Core i7-6700K CPU supported by a Windows 8.1 64-bit environment.

## Results

### Prediction Model Performance Results

As shown in Table 1, the AUC scores ranged between 0.61 and 0.90 for all support needs. Of all models, GBT consistently produced superior AUC scores compared with others, except for predicting emotional support where it ranked second to SVMs. For this reason, we concluded that GBT could most accurately predict a user’s social support need given survey features, and carried out subsequent analyses using this model.

### Feature Analysis Results

**Figure 1:**
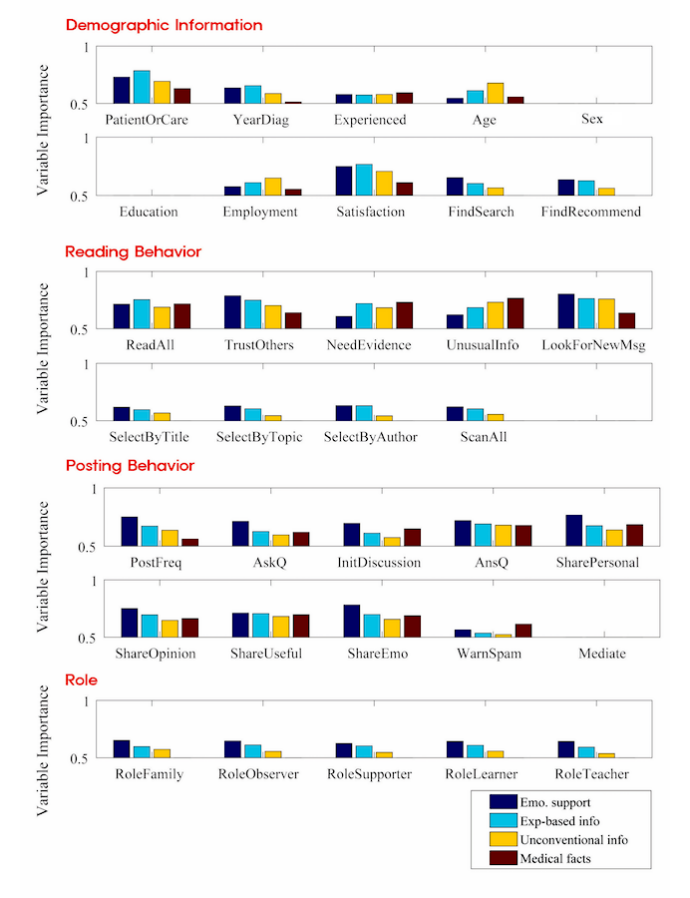
Individual feature importance on each social support need.

Figure 1 shows the results of feature analysis. Each social support needed relies on a distinct set of features for prediction. Again, AUC scores were used to indicate the strength of a feature in its predictability of a user’s social support need. To provide a better understanding on which features are of actual importance, we devoted the rest of this section to interpreting the results of these AUC scores. We also present the individual scores to provide better information (see Multimedia Appendix 2).

Two features out of the Demographic Information category (PatientOrCare and Satisfaction) proved to be significant in predicting social support needs, especially if the user required experience-based information. The high prediction score for experience-based information in the ‘PatientOrCare’ feature indicates that users are more likely to visit OHSNs in search of others’ experiences when they are patients themselves rather than caregivers. The ‘Satisfaction’ feature describes that OHSN users seeking for the fourth social support need, medical facts, have a substantially lower satisfaction level upon using OHSNs compared with other users. Meanwhile, whether a user was introduced to an OHSN through recommendation or Web search did not affect the predictability of any of the 4 social support needs (FindSearch/FindRecommend). Features such as users’ sex, whether someone nearby or a doctor introduced a user to an OHSN, also did not strongly influence the predictability for any of the 4 social support needs.

Features from the Reading Behavior category showed notable characteristics that differ between social support groups. For example, users who trust others were most likely to seek emotional support (TrustOthers), while those who search for evidence in postings were likely to search for experience-based or fact-based information (NeedEvidence).

The ‘Need Evidence’ feature was particularly important in that it functioned differently in predicting emotional and informational support. While searching for evidence was not an important feature for predicting emotional support, it largely affected the predictability of the 3 variants of informational support: experience-based information, unconventional information, and medical facts. Users either searching for the experiences of others or medical facts are bound to be skeptical of what was posted and would carefully look for cues of evidence that support the validity of the posted content. However, this feature was less important to those seeking unconventional information, as for such people the type of information they were searching for may often lack concrete scientific evidence but still be worth knowing.

The Posting Behavior category contained the strongest individual features when it came to predicting emotional support. In fact, 8 of 10 features from this category had the strongest predictability when predicting emotional support needs. Users seeking this social support type were most likely to post frequently (PostFreq), ask questions (AskQ), and share their personal stories and emotions with others (SharePersonal/ShareEmo).

The greatest significance of this category lies in the fact that these features not only help predict which type of social support a user seeks, but even depict how active he/she is as a community member. The number of posts and threads he/she posts rather than reading patterns often determines the activeness of a user within an online community. How frequently one posts (PostFreq), how often one starts up conversations (InitDiscussion), how often one shares opinions with others (ShareOpinion) all serve as measuring sticks. From this perspective, posting behavior can function as an indicator to measure what drives users to actively participate in online communities. Overall, results from this category clearly represent that users of this support need are likely to be the most active users within a community.

The Role category was based on the understanding of users themselves on their self-perceived roles within an OHSN. Contrary to our expectations, features of the Role category did not capture distinguishable characteristics among different social support needs. All role features showed the highest values in predicting users of emotional support.

**Table 1:** Comparison of different classifier models for obtaining AUC values.

	Classification algorithms				
	Gradient boosting tree	Support vector machine	Decision tree	Random forest	Logistic regression
Emotional support	0.87	0.89	0.78	0.85	0.77
Experience-based information	0.86	0.80	0.76	0.83	0.74
Unconventional information	0.80	0.75	0.69	0.75	0.66
Medical facts	0.83	0.72	0.67	0.83	0.61

## Discussion

### Qualitative Analysis on Prediction Results

Prediction scores, AUC scores in this study, show how our model is capable of accurately predicting the 4 social support needs using features from demographic information, reading behaviors, posting behaviors, and self-perceived roles. Using our model, OHSN administrators can categorize their users based on their social support needs. They can better understand whether patients visit their communities in need of emotional support, experience-based knowledge, or other needs, and provide tailored measures to help users fulfill such desires. Our results showed that the posting activities, more so than their perceived roles or demographic information, had high predictability for social support needs.

Our features can be used to identify the characteristics of active users. Likewise, they can also pinpoint the characteristics for user groups that represent inactive and stationary users. Users with the social support need for medical facts closely fit the description of inactive users, having the lowest prediction scores when using “Satisfaction,” “TrustOthers,” “LookForNewMsg,” and “PostFreq” features. Compared with other support needs, users seeking for medical facts were less likely to read new messages (LookForNewMsg), trust what is posted by others (TrustOthers), and post threads on community boards (PostFreq). In sum, users with the social support need of medical facts were less likely to express themselves in OHSNs compared with other users; thus, partially contributing to the retention or user migration problems mentioned earlier. This knowledge can be used to inform OHSN moderators concerned with the activity level within a community.

Our approach discovering the relationship between OHSN usage patterns and social support types is also shown in work by Wang et al. [40]. Predicted user participation levels using posting behaviors and log data to find that companionship within members lead to low attrition rates [41] found that patients with depression frequently seek interaction with others, which lead to improvements in emotional conditions. Yet these studies have not investigated directly predicting how much each user wants a particular social support need. Predictions using data collected from different time periods will help researchers track the transitioning nature of support needs, such as how users in search of medical facts may gradually shift toward providing and seeking emotional support and experience-based knowledge as they become settled within a community.

### Survey Data, User-Generated Data, and User Log Data

Our framework validated through survey data provides a starting point to use user-generated data and log data in OHSNs in predicting social support needs. Even though we used the survey data in this study, we believe that it is worthwhile to compare with other data from different sources. Thus, we compared the advantages and disadvantages of using survey data, user-generated data, and log data in OHSNs for predicting social support needs (see Table 2).

Survey data, as seen from our study, collects direct responses from the OHSN users, such as one’s level of agreement or opinion on a particular characteristic. While surveys can cover all necessary features required for prediction directly from the participants, they are costly in data collection. Response rate and completeness are also challenging factors.

User-generated data include posting behaviors, post contents, and their associated log data; from this data, we can generate total word count, sentiment of the post, and posting frequency. These data, in contrast to the survey data, can be easily collected. OHSNs over different resources allow users to continuously interact with each other and produce contents, which directly represent their imminent needs. Such information can be collected over time via text crawling, but requires expertise on natural language processing and data mining to be applied for prediction.

The last data form, user log data, provides click and page view information, search history, and connection time. The vastness of this type of data affords unique opportunities for new discoveries. Predictions based on the objective datasets, such as user logs, will give more realistic results that reflect the real user intentions compared with survey data. The challenge is that such data are usually difficult to obtain because it requires a proprietary access.

Given that user-generated data and user-log data are unstructured and noisy, our work provides guidelines on what features should researchers focus in considering for accurate prediction. For instance, user postings can be analyzed using natural language processing techniques, such as sentiment analysis and topic modeling to see if the writer requires emotional support. Search history logs can provide information on what types of postings the user is interested in. These processes can boost the prediction performance further and provide more insights.

**Table 2:** Comparison of different data sources for prediction in OHSNs

Data source	Survey data (high-level data)	User-generated data (mid-level data)	User log data (low-level data)
**Effort required to collect data**			
	Design questionnaires	Perform text mining	Extract data from server database
	Conduct surveys	Apply natural language processing on text	
**Data generation rate**			
	Slow	Fast	Instantaneous
	Need to conduct new survey to get recent data	Hundreds of posts written by users everyday	New generated with every user action (eg, access time, search history)
**Interpretability**			
	Very easy to understand	Relatively easy to understand	Difficult to derive meaning from raw data
	Questions directly suited to user’s intentions	Requires data processing to extract features from long texts	Requires insight on what features to obtain from given data
**Data types**			
	Numerical data (eg, scale of 1~10)	Text data (eg, title, user posts, comments)	Periodical data (eg, access time)
	Demographic information (eg, age, sex, region)		Demographic information (eg, user profile information)
	Text data for open-ended questions		Hypertext data (eg, accessed links)
			Text data (eg, keywords typed in for search)
**Obtainable characteristics**			
	A user’s (dis)agreement toward a particular characteristic	Words that represent a user’s main interests or concerns	Visiting frequency
	Open-ended answers toward a question	Response to a particular article	Reading preference
			Search preference

### Limitations and Future Work

Our sample size was relatively small for prediction and our model was based on the survey data, which is due to the low-response rate from the survey recipients [17,42]. Nonetheless, our high-quality data from survey responses in this study can be used as a good example of users’ social needs and other characteristics, which can be predicted using other data sources in future work.

Another potential downfall of surveys is that participants might conceal their true thoughts in fear of being evaluated by others. Although it is challenging to identify the level of honesty in each participant’s responses, we can assume the consistent patterns in our prediction results serve as proof that the majority of survey participants completed their survey truthfully.

Our prediction model was less effective in predicting social support needs using results from open-ended questions (FindSearch, FindRecommend, SelectByTitle, SelectByTopic, SelectByAuthor, ScanAll, and questions from the Role category). Not only were individual prediction scores low compared with multiple-answer questions (see Figure 1), but all prediction results showed a regular pattern of decrease as they moved from predicting emotional support to medical facts.

The low-prediction scores are a result of low participation in answering open-ended questions. Unlike multiple-answer questions, the response rates of open-ended questions were in general under 50% (see Multimedia Appendix 1). Although GBT is capable of using features with missing values [31], one cannot expect significant prediction scores when using features with such handicaps.

Future work should also include expanding our data size by collecting features we found useful in this model from various sources, such as user-generated data and log data. A larger dataset means that cross-validation and other techniques can be applied to further increase accuracy. We can also expand the search scope to patients with acute diseases, who tend to show more information-oriented needs compared with those in chronic conditions.

### Conclusion

We developed a technical framework to predict the social support needs of OHSN users using users’ values and reported OHSN usage patterns based on survey data. We found dominant features that contributed to successful predictions, not only in predicting a user’s desired support needs but also in his/her level of participation. We showed how different granularity of data around OHSN use can be collected and used to make further predictions on OHSN users’ social support needs. We also presented strategies for OHSN administrators to identify the characteristics of users and what values they seek.

Our research contributes to not only understanding the different types of OHSN users but also accurately classifying them according to usage patterns. We thus provide a stepping-stone to understanding what features are found to be important in predicting social support needs and what data sources are realistic in being used as a training data for constructing a prediction model. The value of our methodology lies in assisting administrators and moderators by providing them with guidance on what type of support users can benefit most from. We provide a stepping-stone to improving retention in OHSNs. Our study contributes to OHSN as an intervention tool to improve health behavior and social support.

## Appendix

Multimedia Appendix 1 Survey questions and results.

Multimedia Appendix 2 Area under ROC curve (AUC) scores of individual features and target variables.

Multimedia Appendix 3 Correlation scores of individual features and target variables.

## Abbreviations

AUC: area under receiver operating characteristic curve
OHSN: online health social networks
GBT: gradient boosting tree
ROC: receiver operating characteristic
SVM: support vector machines
