# Supplementary material for: Toward Predicting Social Support Needs in Online Health Social Networks
Source: J Med Internet Res. 2017 Aug 2;19(8):e272. doi: 10.2196/jmir.7660 (PMC5559652; doi:10.2196/jmir.7660)
Supplement: Multimedia Appendix 1 [file jmir_v19i8e272_app1.pdf]

Appendix 1. Survey results

※ Total samples (n=184)

Demographics (8 features)

- **[PatientOrCare]** Are you a patient or a caregiver of the health problem you identified above?

Patient	103 (56.0%)
Caregiver	24 (13.0%)
Other	7 (3.8%)

- **[YearDiag]** How long were you or the patient diagnosed with the health problem identified above?

Less than one year	8 (4.3%)
1~3 years	26 (14.2%)
3~5 years	11 (6.0%)
5~10 years	9 (4.9%)
10~15 years	21 (11.4%)
15~30 years	13 (7.1%)
30 years ~	12 (6.5%)

- **[Experienced]** How much do you feel you are experienced in managing or caring for the health problems identified above?

Very experienced	44 (23.9%)
Somewhat experienced	54 (29.3%)
Little experienced	23 (12.5%)
Not experienced at all	26 (14.1%)

- **[Education]** What is the highest level of education you have completed?

Some high school	37 (20.1%)
Some college	76 (41.3%)
Post graduate	60 (32.6%)

- **[Age]** What is your age?

Under 18 years old	9 (4.9%)
18~29 years old	13 (7.1%)
30~49 years old	62 (33.7%)
50~64 years old	69 (37.5%)
64 years old or older	25 (13.6%)

- **[Gender]** What is your gender?

Male	56 (30.4%)
Female	119 (64.7%)

- **[Employment]** Employment status: are you currently

Employed	93 (50.5%)
Not employed	69 (37.5%)

- **[Satisfaction]** In general, how satisfied are you with the online health support group?

Very satisfied	2 (1.1%)
Satisfied	46 (25%)
Unsure	56 (30.4%)
Dissatisfied	5 (2.7%)
Very dissatisfied	48 (27.1%)

- **[FindSearch/FindRecommend]** Pick one online health support group that you most visit. How did you find out about the support group? *(Open ended)*

Found through search

Yes:	67 (36.4%)
No:	32 (17.4%)

Found through recommendation

Yes:	33 (17.9%)
No:	66 (35.9%)

Reading Behavior (9 features)

Please check how much you agree with the following statements. (*Scale 1 to 5, Strongly Disagree (1), Disagree (2), Neither agree nor disagree (3), Agree (4), Strongly Agree (5))

- **[ReadAll]** I read all messages in online health support groups as much as possible.

Strongly agree	16 (8.7%)
Agree	34 (18.5%)
Neither agree nor disagree	48 (26.1%)
Disagree	30 (16.3%)
Strongly disagree	23 (12.3%)

- **[TrustOthers]** I trust what people post in the online health support groups I go to.

Strongly agree	4 (2.2%)
Agree	31 (16.8%)
Neither agree nor disagree	67 (36.4%)
Disagree	32 (17.4%)
Strongly disagree	14 (7.6%)

- **[NeedEvidence]** I need scientific evidence for me to believe what people post in online health support groups. (3.01 ± 1.17)

Strongly agree	16 (8.7%)
Agree	37 (20.1%)
Neither agree nor disagree	53 (28.8%)
Disagree	25 (13.4%)
Strongly disagree	21 (11.4%)

- **[UnusualInfo]** I look for unconventional information otherwise difficult to find elsewhere. (2.99 ± 1.13)

Strongly agree	12 (6.5%)
Agree	39 (19.6%)
Neither agree nor disagree	53 (28.8%)
Disagree	26 (14.1%)
Strongly disagree	19 (10.3%)

- **[LookForNewInfo]** I regularly visit the online health support groups to look for new messages posted that I haven't read yet. (3.01 ± 1.25)

Strongly agree	20 (10.9%)
Agree	36 (19.6%)
Neither agree nor disagree	42 (22.8%)
Disagree	28 (15.2%)
Strongly disagree	23 (12.5%)

- **[SelectByTitle/SelectByTopic/SelectByAuthor/ScanAll]** How do you select which message to read? *(Open-ended)*

Select by title

Yes:	11 (6.0%)
No:	38 (20.7%)

Select by topic

Yes:	24 (13.0%)
No:	25 (13.6%)

Select by author

Yes:	7 (3.8%)
No:	42 (22.8%)

Scan all

Yes:	10 (5.4%)
No:	39 (21.2%)

Posting Behavior (10 features)

- **[PostFreq]** How often do you post messages in online health support groups? (2.08 ± 1.36)

Everyday	11 (6.0%)
A few times a week	22 (12.0%)
A few times a month	24 (13.0%)
A few times a year	16 (8.7%)
Never	87 (47.3%)

Please answer how often you post in the online health support group with the following intentions. (*Never (1), Rarely (2), Sometimes (3), Often (4), Very Often (5))

- **[AskQ]** I ask questions. (2.19 ± 1.16)

Very often	4 (2.2%)
Often	18 (9.8%)
Sometimes	42 (22.8%)
Rarely	29 (15.8%)
Never	60 (32.6%)

- **[InitDiscussion]** I initiate discussions. (2.10 ± 1.22)

Very often	6 (3.3%)
Often	15 (8.2%)
Sometimes	38 (20.7%)
Rarely	19 (10.3%)
Never	71 (38.6%)

- **[AnswerQ]** I answer others' questions. (2.31 ± 1.32)

Very often	8 (4.3%)
Often	28 (15.2%)
Sometimes	32 (17.4%)
Rarely	19 (10.3%)
Never	65 (35.3%)

- **[SharePersonal]** I share my personal experiences. (2.33 ± 1.34)

Very often	10 (5.4%)
Often	25 (13.6%)
Sometimes	35 (19.0%)

Rarely	18 (9.8%)
Never	65 (35.3%)

- **[ShareOpinion]** I share my opinions. (2.36 ± 1.32)

Very often	10 (5.4%)
Often	26 (14.1%)
Sometimes	33 (17.9%)
Rarely	25 (13.6%)
Never	60 (35.3%)

- **[ShareUseful]** I share useful information. (2.38 ± 1.31)

Very often	7 (3.8%)
Often	31 (16.8%)
Sometimes	33 (17.9%)
Rarely	19 (10.3%)
Never	59 (32.1%)

- **[ShareEmotion]** I share emotional support with other members. (2.39 ± 1.39)

Very often	14 (7.6%)
Often	25 (13.6%)
Sometimes	32 (17.9%)
Rarely	19 (10.3%)
Never	63 (34.2%)

- **[WarnSpam]** I warn about spams and advertisements. (1.93 ± 1.20)

Very often	8 (4.3%)
Often	11 (6.0%)
Sometimes	22 (12.0%)
Rarely	30 (16.3%)
Never	79 (42.9%)

- **[Mediate]** I mediate members to help conversations stay on track. (1.75 ± 1.04)

Very often	2 (1.1%)
Often	10 (5.4%)
Sometimes	25 (13.6%)
Rarely	25 (13.6%)
Never	87 (47.3%)

Role (5 features)

- **[RoleFamily/RoleObserver/RoleSupporter/RoleLearner/RoleTeacher]** How would you describe your role in the online health support group? *(Open ended)*

Role as family member		
	Yes:	29 (15.8%)
	No:	30 (16.3%)
Role as observer		
	Yes:	20 (10.9%)
	No:	39 (21.2%)
Role as supporter		
	Yes:	19 (10.3%)
	No:	40 (21.7%)
Role as learner		
	Yes:	9 (4.9%)

	No:	50 (27.2%)
Role as teacher		
	Yes:	14 (7.6%)
	No:	47 (25.5%)

Values Sought (4 features)

The reason for visiting the online health support group is:
(*Not true at all (1), Not true (2), I don't know (3), True (4), Very true (5))

- **[ExchangeEmo]** To exchange emotional support (3.17 ± 1.32)

Very true	27 (14.7%)
True	42 (22.8%)
I don't know	38 (20.7%)
Not true	18 (9.8%)
Not true at all	25 (13.6%)
Binary	
- Positive	69 (46.0%)
- Negative	81 (54.0%)

- **[HearExp]** To hear about personal experiences from other patients (3.56 ± 1.14)

Very true	29 (15.8%)
True	60 (32.6%)
I don't know	34 (18.5%)
Not true	12 (6.5%)
Not true at all	12 (6.5%)
Binary	
- Positive	89 (60.5%)
- Negative	58 (39.5%)

- **[GetUnusualInfo]** To gain nonmainstream information (3.32 ± 1.19)

Very true	24 (13.0%)
True	48 (26.1%)
I don't know	44 (23.9%)
Not true	16 (8.7%)
Not true at all	16 (8.7%)
Binary	
- Positive	72 (48.6%)
- Negative	76 (51.4%)

- **[SpecificSearch]** To gain information that I am looking for (2.91 ± 1.14)

Very true	12 (6.5%)
True	35 (19.0%)
I don't know	54 (29.3%)
Not true	29 (15.8%)
Not true at all	20 (10.9%)

Binary	
- Positive	57 (33.5%)
- Negative	113 (66.5%)

* (mean ± standard deviation)
