# Supplementary material for: Toward Predicting Social Support Needs in Online Health Social Networks
Source: J Med Internet Res. 2017 Aug 2;19(8):e272. doi: 10.2196/jmir.7660 (PMC5559652; doi:10.2196/jmir.7660)
Supplement: Multimedia Appendix 2 [file jmir_v19i8e272_app2.pdf]

Appendix 2. Area under ROC curve (AUC) scores

	Social support needs (Feature name in dataset)			
	Emotional Support (Exchange Emo)	Experience-based Info (HearExp)	Unconventional info (GetUnusual Info)	Medical facts (Specific Search)
PatientOrCare	0.73	0.79	0.69	0.63
YearDiag	0.64	0.66	0.59	0.51
Experienced	0.58	0.57	0.58	0.59
Age	0.55	0.61	0.68	0.56
Gender	0.51	0.51	0.46	0.47
Education	0.45	0.46	0.42	0.46
Employment	0.58	0.61	0.65	0.55
Satisfaction	0.75	0.77	0.71	0.61
FindSearch	0.65	0.60	0.57	0.47
FindRecommend	0.63	0.63	0.56	0.44
ReadAll	0.71	0.75	0.69	0.72
TrustOthers	0.79	0.75	0.70	0.64
NeedEvidence	0.61	0.72	0.68	0.73
UnusualInfo	0.62	0.68	0.73	0.77
LookForNewMsg	0.80	0.76	0.76	0.64
SelectByTitle	0.62	0.60	0.57	0.47
SelectByTopic	0.63	0.60	0.54	0.46
SelectByAuthor	0.63	0.63	0.54	0.45
ScanAll	0.62	0.60	0.55	0.46
PostFreq	0.75	0.67	0.64	0.56
AskQ	0.71	0.63	0.60	0.62
InitConversation	0.70	0.61	0.57	0.65
AnsQ	0.72	0.69	0.68	0.68
SharePersonal	0.77	0.68	0.64	0.69
ShareOpinions	0.75	0.70	0.65	0.66
ShareUseful	0.71	0.71	0.68	0.70
ShareEmo	0.78	0.70	0.66	0.69
WarnSpam	0.56	0.54	0.52	0.61
Mediate	0.53	0.50	0.46	0.52
RoleActive	0.65	0.60	0.57	0.45
RoleObserver	0.65	0.61	0.56	0.48
RoleSupporter	0.62	0.60	0.55	0.45
RoleLearner	0.64	0.61	0.56	0.47
RoleTeacher	0.64	0.59	0.53	0.46
